# Subtype-Specific m^6^A circRNA Methylation Patterns Identify Epigenetic Biomarker Candidates of Potential Diagnostic and Prognostic Significance in Breast Cancer

**DOI:** 10.3390/ijms27010529

**Published:** 2026-01-04

**Authors:** Amal Qattan, Wafa Alkhayal, Kausar Suleman, Taher Al-Tweigeri, Asma Tulbah

**Affiliations:** 1 Innovation and Research Department, King Faisal Specialist Hospital and Research Centre, Riyadh 11211, Saudi Arabia; 2 College of Medicine, Alfaisal University, Riyadh 11533, Saudi Arabia; 3 Surgical Oncology Department, Cancer Centre of Excellence, King Faisal Specialist Hospital and Research Centre, Riyadh 11211, Saudi Arabia; wkhayal@kfshrc.edu.sa; 4 Medical Oncology Department, Cancer Centre of Excellence, King Faisal Specialist Hospital and Research Centre, Riyadh 11211, Saudi Arabia; ksuleman@kfshrc.edu.sa (K.S.); ttwegieri@kfshrc.edu.sa (T.A.-T.); 5 Pathology and Laboratory Medicine Department, King Faisal Specialist Hospital and Research Centre, Riyadh 11211, Saudi Arabia; tulbah@kfshrc.edu.sa

**Keywords:** triple-negative breast cancer, non-coding RNA, circRNA, microRNA, m^6^A methylation, epitranscriptomics, post-transcriptional regulation, biomarker, therapeutic target, precision oncology, targeted therapy, tumor progression

## Abstract

Breast cancer subtypes are known to have important pathobiological and clinical features. For example, triple-negative breast cancer (TNBC) remains one of the most aggressive and treatment-resistant breast cancer subtypes, lacking hormone and HER2 targets. Increasing evidence suggests that circular RNAs (circRNAs) and their N6-methyladenosine (m^6^A) modifications play critical roles in cancer biology through the regulation of gene expression, stability, and signaling networks. This study aimed to identify m^6^A methylation patterns in circRNAs among breast cancer subtypes, explore their potential biological functions, and assess their diagnostic and prognostic relevance compared with luminal breast cancer subtypes. Genome-wide profiling of m^6^A-modified circRNAs was conducted in TNBC and luminal breast tumor samples using methylated RNA immunoprecipitation followed by microarray analysis. Differential methylation and expression analyses were integrated with pathway enrichment, survival correlation, and receiver operating characteristic (ROC) curve assessments to identify subtype-specific and clinically relevant circRNA candidates. Distinct m^6^A circRNA methylation signatures were identified across breast cancer subtypes, with TNBC showing enrichment in pathways related to Wnt/β-catenin, CDC42 GTPase signaling, and cytoskeletal remodeling. Several circRNAs, including those derived from ZBTB16, DOCK1, METTL8, and VAV3, exhibited significant hypermethylation and high diagnostic accuracy (AUC > 0.80). Survival analyses revealed associations between circRNAs from key host genes and overall or relapse-free survival, suggesting prognostic potential. These findings uncover subtype-specific m^6^A circRNA methylation landscapes that may contribute to tumor aggressiveness and heterogeneity. Identified circRNAs represent candidates for investigation as biomarkers for subtype classification and prognosis and may inform future research into epigenetic and post-transcriptional therapeutic targets in breast cancer.

## 1. Introduction

Breast cancer subtypes are defined according to gene expression profiles and are routinely classified by immunohistochemistry in clinical practice [1]. Such classification has important implications for treatment selection. Triple-negative breast cancer (TNBC) accounts for 10–20% of all breast cancers and is characterized by the absence of estrogen receptor, progesterone receptor, and HER2 amplification, leading to poorer prognoses compared to other subtypes [2,3]. TNBC disproportionately affects younger women and certain racial/ethnic groups, with higher rates of recurrence and distant metastasis contributing to elevated mortality [1,4]. Unlike hormone receptor-positive or HER2-positive breast cancers, TNBC lacks targeted therapies, leaving chemotherapy as the mainstay of treatment and underscoring the urgent need for novel biomarkers and therapeutic strategies [5,6].

Pathophysiologically, TNBC differs substantially from luminal breast cancers, with basal-like molecular features, higher proliferative indices, and enhanced genomic instability [7,8]. TNBC tumors display activation of oncogenic signaling pathways, including those mediated by Rho GTPases such as CDC42 and RAC1, which regulate cell polarity, invasion, and metastasis [9,10]. By contrast, luminal subtypes are largely driven by hormone receptor signaling, which provides both diagnostic and therapeutic targets absent in TNBC [9,11]. These molecular differences highlight the distinct clinical course and therapeutic challenges of TNBC relative to luminal cancers. In 2016, Lehmann et al. introduced a classification that further categorizes TNBC into several subtypes: basal-like 1 (BL1), basal-like 2 (BL2), immunomodulatory (IM), mesenchymal (M), mesenchymal stem-like (MSL), and luminal androgen receptor (LAR) [12].

**Figure 1:**
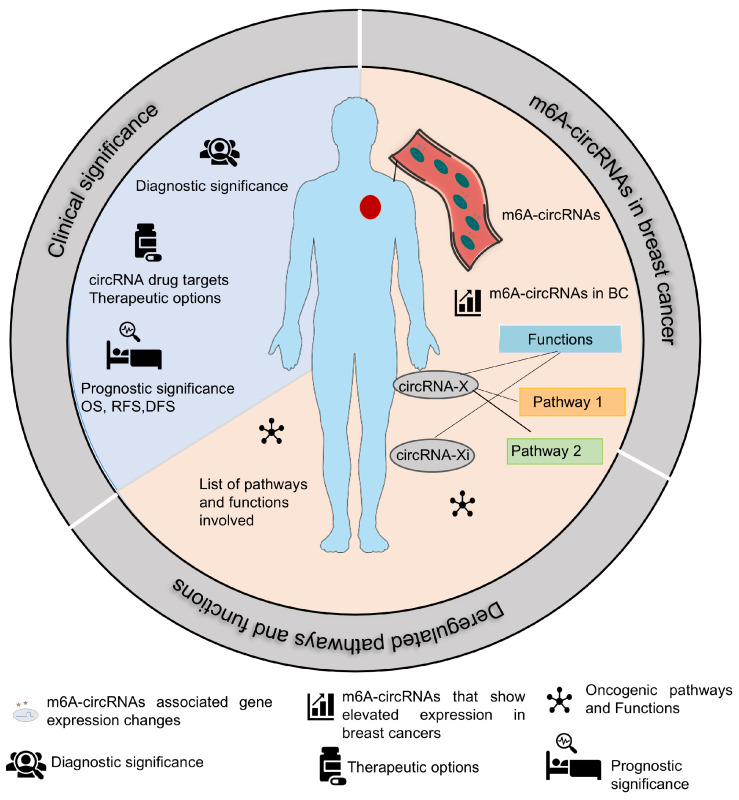
Diagnostic, prognostic, therapeutic, and clinical significance of m^6^A-modified circRNAs in breast cancer. This schematic illustrates the integrated analytical framework in this study to identify breast cancer subtype–specific m^6^A-modified circRNAs and to link them with associated oncogenic pathways, biological functions, and clinical significance, including diagnostic relevance, prognostic value (OS, RFS, DFS), and potential therapeutic implications.

Circular RNAs (circRNAs) are non-coding RNAs that regulate gene transcription and splicing, bind miRNA, act as sponges, and can even encode small peptides [13,14]. Their expression, usually from protein-coding genes, is specific to tissues and cells and can mediate physiological and pathophysiological functions [15,16,17]. Emerging evidence suggests that circRNAs play central roles in breast cancer biology through their function as competing endogenous RNAs (ceRNAs) that modulate microRNA availability and downstream oncogenic signaling [18,19]. These circRNAs commonly affect the stability and expression of mRNAs transcribed from their host gene since they are frequently derived from the exonic sequences of these genes [18]. CircRNAs have been shown to regulate angiogenesis, proliferation, and invasion in multiple cancers, including TNBC, by sponging microRNAs and interacting with proteins that control gene expression [18,20]. Importantly, circRNAs exhibit stability, tissue specificity, and detectability in biofluids, making them candidates as potential diagnostic and prognostic biomarkers in breast cancer [21,22] (Figure 1).

In addition to their expression, circRNAs undergo post-transcriptional regulation via N6-methyladenosine (m^6^A) modification, which influences their stability, translation potential, and interactions with binding partners [23,24]. N6-methyladenosine (m^6^A) is the most abundant internal RNA modification found in both messenger RNAs and non-coding RNAs, including circRNAs [25,26].

Placed by methyltransferases such as METTL3 and METTL14 and removed by demethylases like FTO and ALKBH5, m^6^A dynamically regulates RNA fate and function [27]. In circRNAs, m^6^A influences stability, cytoplasmic export, and interactions with microRNAs or RNA-binding proteins, thereby modulating their function as microRNA sponges and affecting cancer-related signaling [28,29]. Increased m^6^A methylation on circRNAs enhances their stability, promotes nuclear export, and facilitates binding to reader proteins, thereby strengthening their roles as microRNA sponges or enabling peptide translation when open reading frames are present. This modification can improve recognition by translation machinery, leading to higher circRNA abundance and potential protein-coding capacity. Conversely, m^6^A hypomethylation decreases circRNA stability and may alter subcellular localization, often resulting in nuclear retention or degradation. Reduced interaction with stabilizing reader proteins under hypomethylation can impair the circRNA’s regulatory functions, diminishing its influence on microRNA binding and protein interactions. This modification also helps the cell distinguish endogenous circRNAs from foreign RNA species, contributing to immune regulation [27]. Because m^6^A-modified and unmodified forms of the same circRNA can exhibit distinct behaviors, quantifying their modification stoichiometry provides crucial insights into their biological roles and impact on disease [25,26].

Crosstalk between circRNAs and m^6^A regulators has been implicated in tumor progression and therapy resistance, including in breast cancer [30,31]. Genome-wide profiling has revealed widespread and cell-type-specific m^6^A methylation of circRNAs, but the functional significance of these patterns in TNBC remains poorly understood [32,33]. This knowledge gap underscores the need to dissect subtype-specific circRNA methylation landscapes.

Given TNBC’s aggressive phenotype, elucidating how m^6^A-modified circRNAs contribute to its pathophysiology may yield both mechanistic and clinical insights. Prior studies have demonstrated that circRNAs regulate key pathways such as Wnt, PI3K/AKT, and EMT in TNBC, often through ceRNA activity [34,35]. Altered m^6^A modification could modulate these effects by controlling circRNA expression or sponging capacity, thereby shaping oncogenic signaling networks [36,37]. Identifying TNBC-specific m^6^A methylation signatures in circRNAs may thus clarify the molecular basis of subtype differences and reveal candidate therapeutic targets [38,39].

The present study aims to characterize subtype-specific alterations in m^6^A methylation of circRNAs in breast cancers. Specifically, we seek to (i) define subtype-specific circRNA methylation signatures, (ii) identify associated pathological functions to facilitate mechanistic studies and therapeutic discovery, and (iii) evaluate associations with clinical outcomes to nominate candidate prognostic markers. By integrating circRNA methylation with functional and clinical data, this work aims to advance understanding of breast cancer biology and open avenues for subtype-specific diagnostics and therapies [40,41].

## 2. Results

### 2.1. Identification of Differentially m^6^A-Modified circRNAs Between TNBC and Luminal Subtypes

**Figure 2:**
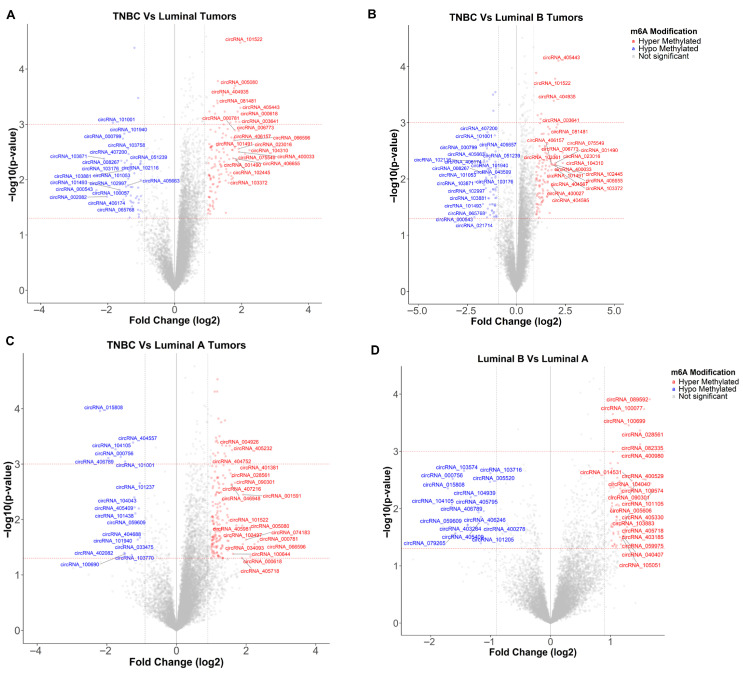
Volcano plots showing differentially m^6^A-modified circRNAs across distinct breast cancer subtypes: (**A**) TNBC vs. Luminal tumors, (**B**) TNBC vs. Luminal B tumors, (**C**) TNBC vs. Luminal A and (**D**) Luminal B vs. Luminal A tumors. Each plot displays the log_2_ fold change on the x-axis against the −log_10_ adjusted *p*-value on the y-axis for m^6^A modification differences. Red dots denote hypermethylated circRNAs, blue dots denote hypomethylated circRNAs, and gray dots indicate circRNAs with non-significant changes. Top 20 circRNAs hyper- or hypomethylated in the comparisons within the significant (adjusted *p* < 0.05 and |log_2_ FC| > 2) are labeled.

Fresh breast cancer tissues were collected from eligible patients undergoing surgical resection in our hospital prior to the administration of any adjuvant therapies. Patient characteristics are described in Supplementary Table S1. Samples were subjected to MeRIP and circRNA Epitranscriptomics analysis. Volcano plots comparing m^6^A methylation profiles of circRNAs across breast cancer subtypes revealed distinct subtype-specific alterations. Each comparison (TNBC vs. Luminal tumors, TNBC vs. Luminal B, TNBC vs. Luminal A, and Luminal B vs. Luminal A) displays the log_2_ fold change in methylation levels on the x-axis and the −log_10_ adjusted *p*-value on the y-axis, highlighting significantly altered circRNAs (*p* < 0.05, |fold change| > 2) (Figure 2; Supplementary Table S2). Red points denote hypermethylated circRNAs, blue points indicate hypomethylated circRNAs, and gray points represent non-significant changes. The top 20 up- and downregulated circRNAs are annotated, illustrating that m^6^A modification patterns distinguish TNBC from luminal subtypes and reflect the potential epigenetic signatures of underlying tumor aggressiveness.

**Figure 3:**
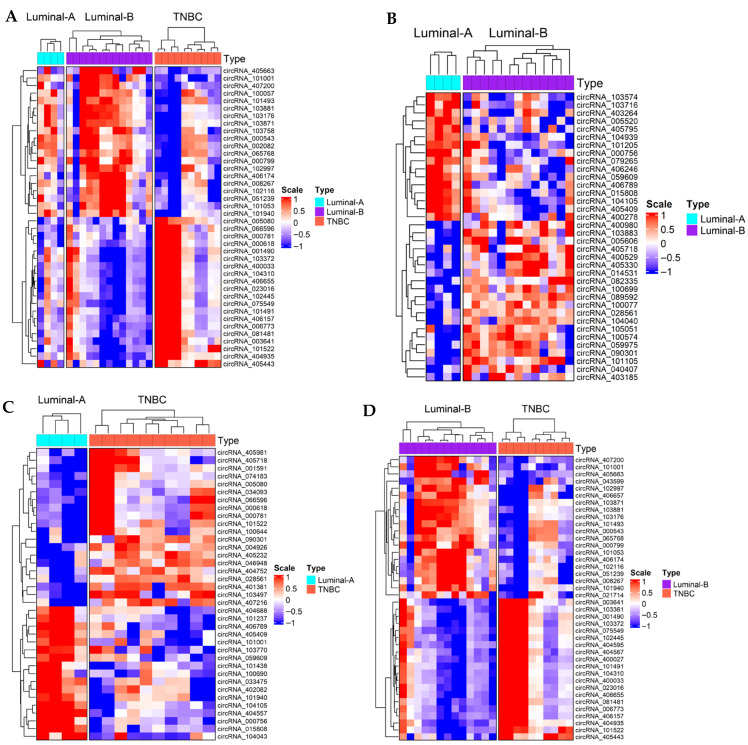
Heatmap showing the methylation patterns of the top 20 differentially m^6^A-modified circRNAs across breast cancer subtypes. The heatmaps depict the methylation pattern of circRNAs that exhibited significant differential m^6^A modifications among the comparisons: (**A**) TNBC vs. Luminal Tumors, (**B**) Luminal A vs. Luminal B tumors, (**C**) Luminal A vs. TNBC and (**D**) Luminal B vs. TNBC. Each row represents individual circRNA, and columns correspond to tumor samples grouped by breast cancer subtype. Color intensity reflects the relative m^6^A methylation level, with red indicating higher methylation and blue indicating lower methylation. Hierarchical clustering of both circRNAs and samples was performed to highlight subtype-specific methylation patterns. Significantly hyper- or hypomethylated circRNAs with significant *p*-value range < 0.05 and |FC| >2 were considered differentially expressed. Columns are split according to predefined breast cancer subtypes (Luminal A, Luminal B or TNBC) using the “column_split” argument of ComplexHeatmap package (version 2.18.0); hence, the samples in the column dendrograms are not linked across tumor types. Sample names were omitted in the heatmap for clarity, as subtype identity is already indicated in the annotation bar.

Among the most significantly altered circRNAs, hsa_circRNA_404935, derived from the ZBTB16 gene, showed strong hypermethylation (FC = 3.42; log_2_ FC = 1.77; *p* = 2.23 × 10^−4^) and excellent discriminatory power (AUC = 0.88) between TNBC and luminal tumors, suggesting its potential as a candidate biomarker for aggressive disease. Conversely, hsa_circRNA_404557, originating from VAV3, exhibited significant hypomethylation (FC = 0.42; log_2_ FC = −1.25; *p* = 4.09 × 10^−4^; AUC TNBC vs. Luminal = 1.0), distinguishing TNBC from both Luminal A and B tumors. These patterns underscore the functional relevance of m^6^A regulation in circRNA biology and its contribution to TNBC pathophysiology. Heatmaps for comparisons of the top 20 differentially methylated circRNAs between breast cancer subtypes were generated, grouping tumors by subtype (Figure 3 and Supplementary Figure S4). A heatmap including all circRNAs identified as differentially methylated is included in Supplementary Figure S1. Collectively, these results indicate differential epitranscriptomic profiles in breast cancer, identifying m^6^A-modified circRNAs as promising candidates for subtype-specific biomarkers. The differences observed among TNBC samples in the heatmap may reflect underlying molecular heterogeneity within TNBC, potentially indicative of distinct biological subgroups. However, validation in larger cohorts is required.

**Figure 4:**
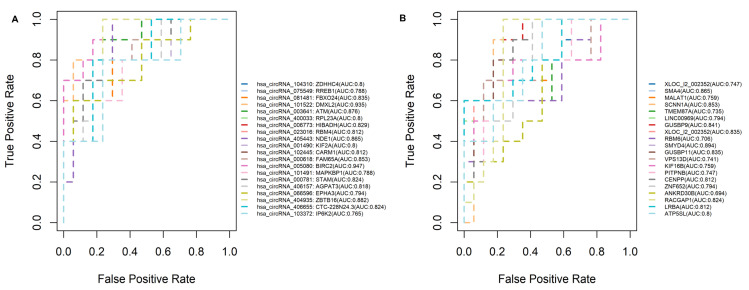
Receiver operating characteristic (ROC) curve analysis of circRNA m^6^A modifications distinguishing TNBC from Luminal breast cancer subtypes. ROC analysis was performed to assess the discriminatory power of the top 20 differentially m^6^A-modified circRNAs between triple-negative breast cancer (TNBC) and Luminal tumors, shown separately for (**A**) hypermethylated and (**B**) hypomethylated categories, with True Positive Rate (Sensitivity) on Y-axis against the False Positive Rate (1-specificity) on X-axis. The ROC curves illustrate the sensitivity and specificity of these circRNAs in classifying the two subtypes. The area under the curve (AUC) was calculated to quantify diagnostic performance. Higher AUC values reflect greater subtype specificity. The corresponding AUC values of each circRNA, together with their host genes, are displayed on the right side of the plot.

Receiver operating characteristic (ROC) curve analysis of circRNA m^6^A modifications distinguishing TNBC from Luminal breast cancer subtypes was conducted. ROC analysis was performed to assess the discriminatory power of the top 20 differentially m^6^A-modified circRNAs between TNBC and Luminal tumors. The resulting AUCs demonstrated the utility of circRNAs to distinguish TNBC from Luminal subtypes through detection of differential m^6^A modification patterns (Figure 4). Several circRNAs identified in our analysis, including hsa_circRNA_104310 (ZDHHC4, AUC = 0.80), hsa_circRNA_404935 (ZBTB16, AUC = 0.882), hsa_circRNA_003641 (ATM, AUC = 0.876), and hsa_circRNA_102445 (CARM1, AUC = 0.812), demonstrated strong discriminatory power across breast cancer subtypes (Figure 4A,B and Supplementary Table S3).

**Figure 5:**
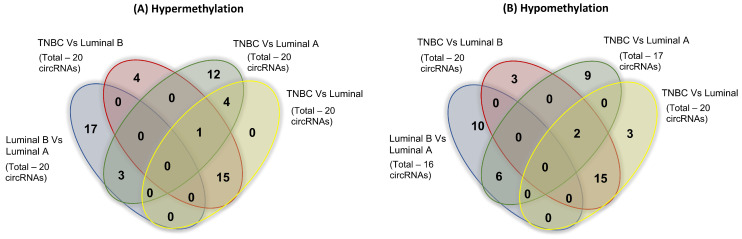
Venn diagrams illustrating the overlap of differentially m^6^A-modified circRNAs across breast cancer subtype comparisons. (**A**) Hypermethylated circRNAs and (**B**) Hypomethylated circRNAs identified from pairwise comparisons between TNBC vs. Luminal A, TNBC vs. Luminal B, TNBC vs. all luminal subtypes, and Luminal A vs. Luminal B. Numbers indicate the counts of unique and shared circRNAs across the comparison groups.

Overlap among circRNAs in different comparisons (e.g., TNBC vs. Luminal A, TNBC vs. Luminal B, etc.) indicates that certain circRNAs consistently undergo differential m^6^A methylation across multiple breast cancer subtypes (Figure 5 and Supplementary Figure S2 and Supplementary Table S4). We identified several TNBC-specific circRNA methylation alterations that were consistent among comparisons with all luminal, luminal A, and luminal B tumors. Those that were hypomethylated included hsa_circRNA_100690 (host gene, GFRA1), hsa_circRNA_101940 (SMYD4), hsa_circRNA_101001 (SCNN1A), hsa_circRNA_402082 (ZNF91), and hsa_circRNA_404557 (VAV3). Notably, VAV3 is associated with angiogenesis and the CDC42 GTPase cycle in TNBC [42]. Those that were hypermethylated included hsa_circRNA_091761 (BCAP31), hsa_circRNA_103497 (SLC33A1), hsa_circRNA_074183 (MATR3), hsa_circRNA_402697 (TOP3B), hsa_circRNA_000781 (STAM), hsa_circRNA_025593 (LDHB), hsa_circRNA_057041 (METTL8), hsa_circRNA_101522 (DMXL2), hsa_circRNA_001278 (GABPA), hsa_circRNA_066596 (EPHA3), hsa_circRNA_005080 (BIRC2), hsa_circRNA_000618 (FAM65A), and hsa_circRNA_034093 (NIPA1). Notably, METTL8 is a methylase involved in epitranscriptomic regulation in cancer, tumor cell migration, and tumorigenesis [43].

### 2.2. Analysis of Subtype-Specific m^6^A-Modified circRNA Host Genes

#### 2.2.1. Genomic Context

**Figure 6:**
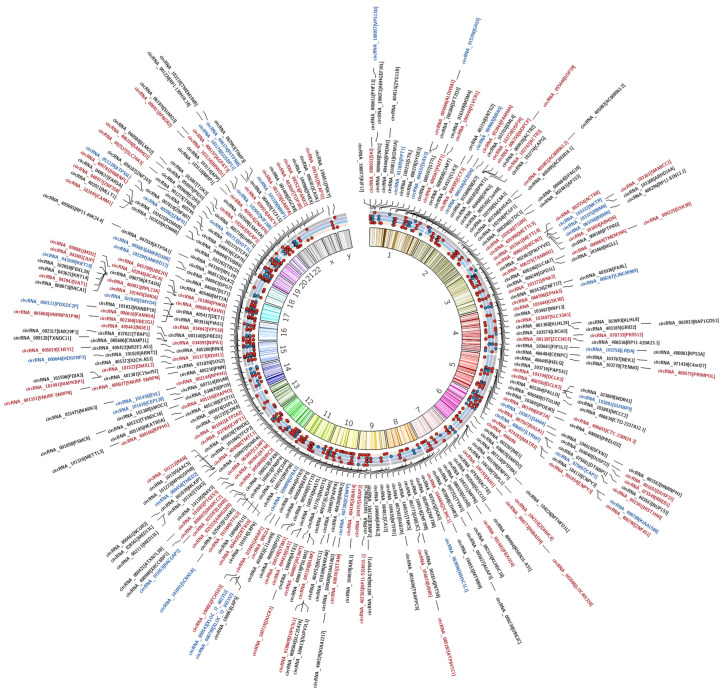
Circos plot depicting the genomic distribution of m^6^A modification patterns across breast cancer subtypes. The plot illustrates the genome-wide chromosomal distribution of circRNAs along human chromosomes. The inner chromosome ideogram displays chromosome numbers and boundaries arranged in a circular layout, serving as a genomic reference, while the outer tracks represent circRNAs harboring m^6^A modifications distributed across the genome.

Differentially m^6^A-modified circRNAs across breast cancer subtypes were mapped to chromosomal locations using a Circos plot to visualize an integrated overview of the data collected and the m^6^A circRNA landscape in breast cancer (Figure 6). This genomic analysis reveals a complex network of interactions among circRNAs with differential methylation between subtypes, with prominent connections centered around regions on chromosomes 1, 3, 11, 12, 15, and 17.

Each dot corresponds to an individual circRNA. The color of each scatter point represents the direction of modification (red = hypermethylated; blue = hypomethylated). CircRNAs differentially modified in at least one comparison are labeled in the outer text ring. circRNAs differentially modified from TNBC vs. Luminal comparisons are additionally annotated, with red denoting hypermethylation and blue denoting hypomethylation in TNBC. Collectively, these tracks provide an integrated view of chromosomal enrichment patterns and reveal genomic regions exhibiting subtype-specific circRNA m^6^A modification dynamics.

**Figure 7:**
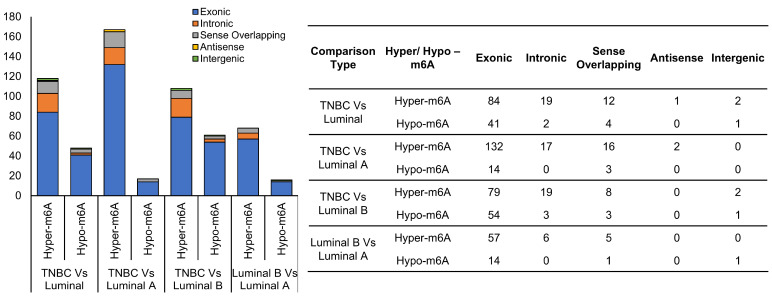
Differentially m^6^A-modified circRNA categories based on genomic loci. The stacked bar diagram shows the distribution of circRNA categories (Exonic, Intronic, Sense Overlapping, Antisense, Intergenic) among differentially m^6^A-modified circRNAs for the comparisons: (1) TNBC vs. Luminal, (2) TNBC vs. Luminal A, (3) TNBC vs. Luminal B, and (4) Luminal B vs. Luminal A. Each comparison includes both hyper-m^6^A and hypo-m^6^A circRNAs. Most differentially m^6^A-modified circRNAs originate from exons, followed by introns, with smaller proportions from other genomic regions.

circRNAs originating from different genomic regions can be derived from distinct splicing or circularization mechanisms [44,45,46]. Different circRNA origins may indicate unique roles, e.g., exonic circRNAs often act as microRNA sponges or regulators of translation. We therefore analyzed the genomic context of the host genes of differentially m^6^A-modified circRNAs. Host genes of m^6^A circRNAs with differing methylation between subtypes were categorized as exonic, intronic, sense-overlapping, antisense, or intergenic and plotted in Figure 7.

#### 2.2.2. Molecular Pathway Enrichment Analysis

**Figure 8:**
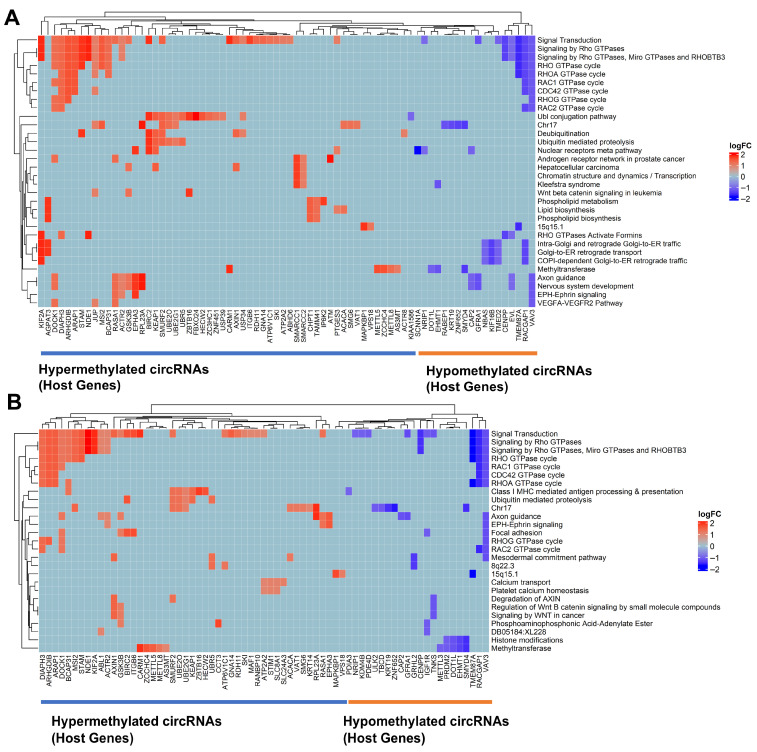
Enrichment analysis of host genes for differentially m^6^A-modified circRNAs. Host genes corresponding to differentially m^6^A-modified circRNAs were identified, and DAVID functional annotation analysis was performed to determine significantly enriched biological and functional terms (*p*-value < 0.05). Using the R package GOplot, relationships between enriched terms and associated host genes are visualized as heatmaps for the comparisons: (**A**) TNBC vs. Luminal tumors, (**B**) TNBC vs. Luminal B tumors. Each column represents the log2 fold change (logFC) of m^6^A methylation for a circRNA with its corresponding host gene on the x-axis. Red indicates higher logFC (hypermethylated), blue indicates lower logFC (hypomethylated), and light blue indicates no significant change or absence of association for a given gene-term pair. Hierarchical clustering groups genes with similar annotations and methylation patterns, highlighting functionally related sets of hyper- and hypomethylated circRNAs.

Host genes corresponding to differentially m^6^A-modified circRNAs were identified, and DAVID functional annotation analysis was performed to determine significantly enriched (*p*-value < 0.05) biological and functional terms. Using the R package GOplot (version 1.0.2), the relationships between enriched terms and associated host genes of circRNAs are visualized as heatmaps for TNBC versus Luminal A and TNBC versus Luminal B comparisons in Figure 8. Other subtype comparisons are included in Supplementary Figure S3.

In TNBC, we observed key Wnt signaling-related host genes in the hypermethylated category. For example, those targeting GSK3B were hypermethylated and can lead to the activation of Wnt signaling. Also, we observed dysregulation of circRNAs involved in non-canonical Wnt signaling pathway activation, which can lead to the further activation of RHOA-GTPAse signaling.

CDC42 GTPase cycle was significantly enriched among TNBC-specific differences in m^6^A methylation of circRNAs and their interactions with specific microRNAs (miRNAs) (Figure 8, Supplementary Table S5). Several circRNAs derived from key host genes related to this signaling pathway display contrasting methylation. For example, circRNAs from RACGAP1 (hsa_circRNA_101053) and VAV3 (hsa_circRNA_404557) are hypomethylated, with Fold Change (FC) values of 0.3824 and 0.4345, respectively, and are downregulated, with fold changes of 0.2311 and 0.1276. Conversely, circRNAs from ARAP1 (hsa_circRNA_023461), ARHGDIB (hsa_circRNA_025522), JUP (hsa_circRNA_043602), and DIAPH3 (hsa_circRNA_405156) are hypermethylated, with FC values exceeding 2.0.

Enrichment analysis highlighted key molecular changes in circRNAs that may influence angiogenesis and tumor progression, particularly the VEGFA-VEGFR2 pathway in TNBC (Figure 8, Supplementary Table S5). VAV3 circRNA (hsa_circRNA_404557) hypomethylation (FC 0.4346, *p* = 4.13 × 10^−5^) was a significant observation related to this pathway. RASA1 (hsa_circRNA_007507) and JUP (hsa_circRNA_043602), which sequester specific miRNAs that modulate the VEGFA-VEGFR2 pathway, are hypermethylated and upregulated. DOCK1 (hsa_circRNA_100719), also acting as an miRNA sponge (miR-936) in angiogenesis pathways [47], is hypermethylated and upregulated.

We identified several TNBC-specific alterations in methyltransferase enzymes related to cancer pathogenesis, such as SMYD4 (involved in breast cancer progression) [48,49], EHMT1, DOT1L, Zinc Finger CCHC-Type Containing 4 (ZCCHC4), a modifier of 28S ribosomal RNA (rRNA) with a crucial role in mRNA translation and cancer chemoresistance [50,51], METTL8, CARM1, AS3MT, and METTL5 (Figure 8, Supplemental Table S5).

These findings identify critical pathogenic processes that are associated with TNBC-specific alterations in circRNA methylations. This provides rationale for the investigation of these circRNAs as potential therapeutic targets and biomarkers of prognosis.

### 2.3. Association of Subtype-Specific circRNAs with Survival in TNBC

**Figure 9:**
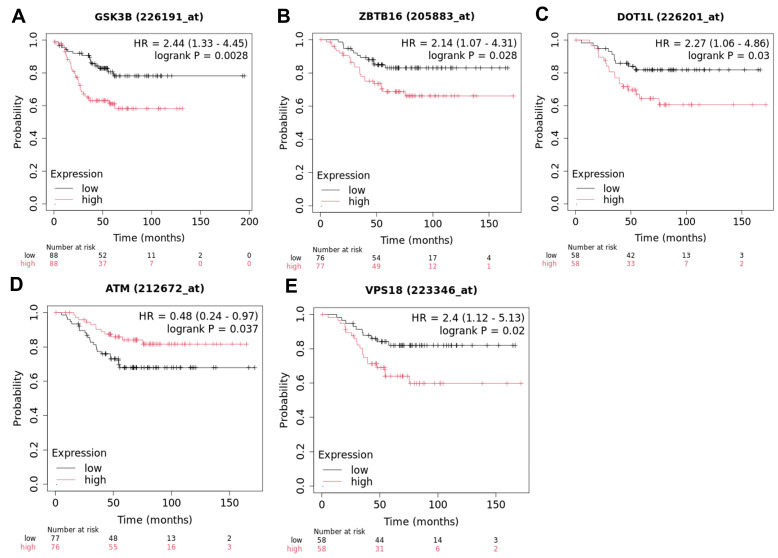
Kaplan–Meier survival analyses of host genes encoding differentially m^6^A-methylated circRNAs. (**A**) Recurrence-free survival (RFS) stratified by GSK3B expression. (**B**–**E**) Overall survival (OS) stratified by expression of ZBTB16, DOT1L, ATM, and VPS18, respectively. Patients were divided into high- and low-expression groups, and survival differences were assessed using the log-rank test. Hazard ratios (HRs) with 95% confidence intervals are shown.

To identify m^6^A-altered circRNAs discovered in our analysis that are related to disease progression and prognosis, we investigated TNBC-specific host genes that we identified using public datasets and Kaplan–Meier analyses. Survival analyses identified that several host genes of circRNAs with TNBC-specific alterations in m^6^A methylation were significantly linked to overall survival (OS) or recurrence-free survival (RFS) in TNBC (Figure 9, Supplementary Table S6).

A pathophysiologically relevant host gene that was associated with recurrence-free survival was GSK3B. Increased expression of GSK3B correlated with a higher recurrence risk (HR = 2.44, 95% CI 1.33–4.45; *p* = 0.0028). Elevated expression of ZBTB16 was associated with worse overall survival (HR = 2.14, 95% CI 1.07–4.31; *p* = 0.028). Its circRNA (hsa_circRNA_404935) was hypermethylated in our analysis. Higher expression of DOT1L was associated with poor prognosis (HR = 2.27; 95% CI 1.06–4.86; *p* = 0.03). The associated circRNA (hsa_circRNA_405713) was hypomethylated in TNBC in our analysis. In contrast, elevated ATM expression was protective (HR = 0.48; 95% CI 0.24–0.97; *p* = 0.037). Its circRNA (hsa_circRNA_003641) was hypermethylated in TNBC. Higher expression of the hypermethylated circRNA (hsa_circRNA_034642), linked to VPS18 upregulation, was associated with poor survival (HR = 2.4; 95% CI 1.12–5.33; *p* = 0.02).

## 3. Discussion

### 3.1. Subtype-Specific Methylation Patterns

Our analysis revealed several significant differences in m^6^A-methylated circRNAs between breast cancer subtypes. Many of these have known pathophysiological functions in breast cancer. For example, (hsa_circRNA_101522, Chr.15, exonic (DMXL2)), whose host gene DMXL2 is reported to drive EMT and promote endocrine therapy resistance in breast cancer through Notch pathway hyperactivation [52], was found to be specifically upregulated among the top 20 circRNAs in three comparisons: TNBC vs. Luminal, TNBC vs. Luminal A, and TNBC vs. Luminal B.

Similarly, circRNAs derived from genes associated with cell cycle progression, mitosis, and chromosomal segregation, such as KIF2A (hsa_circRNA_001490, Chr.5, exonic), FBXO24 (hsa_circRNA_081481, exonic, Chr.7), and RPL23A [53,54,55], as well as those involved in transcriptional and epigenetic regulation, such as CARM1 and ZBTB16 [56,57], and signaling pathway effectors, such as RREB1, IP6K2, and MAPKBP1) [58,59,60], were consistently observed among the top 20 circRNAs in TNBC vs. Luminal and TNBC vs. Luminal B comparisons (Figure 2, Supplementary Table S2). This suggests that TNBC exhibits differential hyper-m^6^A methylation modifications in these regulatory circRNAs compared with Luminal subtypes.

In addition, other prominent circRNAs are derived from host genes such as BIRC2, STAM, FAM65A, and EPHA3, which are known to mediate apoptosis resistance, receptor signaling, cytoskeletal remodeling, and cell–cell communication, respectively [61,62,63,64], were found to be hyper-m^6^A modified in TNBC compared to Luminal A tumors.

### 3.2. AUC Analysis and Discrimination Between Subtypes

Certain circRNAs identified in our analysis demonstrated exceptionally strong discriminatory power across breast cancer subtypes, e.g., hsa_circRNA_104310 (host gene: ZDHHC4), hsa_circRNA_404935 (ZBTB16), hsa_circRNA_003641 (ATM), and hsa_circRNA_102445 (CARM1) (Figure 4A,B, Supplementary Table S3). Their distinct m^6^A methylation profiles reflect subtype-specific epitranscriptomic regulation and appear to correlate with potential influence on tumor progression. The consistently high AUC values suggest that these methylation markers could support more precise molecular classification of breast cancer, particularly in distinguishing TNBC from luminal subtypes.

These findings underscore the broader biological significance of circRNA methylation in defining the heterogeneity of breast cancer. Hypermethylation of specific circRNAs, such as those associated with ZBTB16 and CARM1, may contribute to aggressive phenotypes by stabilizing transcripts or enhancing oncogenic signaling, while others, like ATM-related circRNAs, could indicate protective or DNA repair-linked functions. Collectively, these subtype-specific m^6^A signatures highlight circRNA methylation as a promising potential class of epigenetic biomarkers and therapeutic targets, justifying further investigation. Targeting the m^6^A methylation machinery to modulate circRNA function could open new avenues for precision medicine in breast cancer, warranting further validation of these candidates in larger patient cohorts and metastatic TNBC (mTNBC) populations.

Overlap, or lack thereof, of differentially methylated circRNAs between subtypes, as shown in the Venn diagram in Figure 5, may be useful in diagnostics to identify subtypes. Those circRNAs that are observed in TNBC vs. Luminal B and not in TNBC vs. Luminal A tumors may be used to distinguish the Luminal B and Luminal A subtypes. In other words, subtype-specific circRNA modifications can be identified.

Among genes whose circRNAs were universally altered in TNBC compared to other subtypes, VAV3 is an example of a TNBC-specific alteration with both diagnostic potential, due to specificity, and pathophysiological relevance. As a guanine nucleotide exchange factor, VAV3 relates to the pathophysiology of triple-negative breast cancer (TNBC) by promoting cytoskeletal remodeling and metastatic potential through activation of Rho family GTPases. VAV3 facilitates ERBB4-mediated cancer cell migration, linking growth factor signaling to enhanced motility and invasion [65]. Its dysregulation may also intersect with metabolic and exosomal pathways known to drive TNBC aggressiveness. Exosome-mediated communication has been shown to influence metastatic behavior and immune evasion [66], while metabolic reprogramming through the mevalonate pathway, regulated by VAV3, supports tumor growth and distant spread [42]. Collectively, these findings position VAV3 as a potential key mediator of TNBC progression, bridging signaling, cytoskeletal dynamics, and metabolic adaptation in aggressive breast cancer phenotypes.

Others, including DOCK1 and ZBTB16, have both pathophysiologic function and relevance to disease progression and outcomes as discussed below. Our findings that m^6^A methylation of DOCK1-derived circRNA is increased in TNBC compared to luminal subtypes align with evidence that DOCK1 promotes aggressive breast cancer behavior through its regulation of cell motility, invasion, and epithelial–mesenchymal transition (EMT). DOCK1, a Rac-specific guanine nucleotide exchange factor, drives metastatic signaling downstream of HER2 and is essential for Rac1-mediated cytoskeletal remodeling and invasion [67]. Elevated DOCK1 expression enhances tumor cell migration via pathways involving RRP1B and Claudin-1, particularly in claudin-low and triple-negative breast cancer cells [68]. Additionally, DOCK1 silencing or inhibition suppresses invasion by reducing RhoA/Rac1 pathway activation [69], while miR-486-5p has been shown to inhibit IL-22-induced EMT by directly repressing DOCK1 [70]. The increased m^6^A methylation observed in DOCK1 circRNA may therefore enhance its stability or translation, amplifying these oncogenic pathways and contributing to the heightened invasiveness and poor prognosis characteristic of TNBC [71].

Our observation that m^6^A methylation of ZBTB16-derived circRNA is increased in TNBC suggests that this modification may alter the gene’s typical tumor-suppressive functions, contributing to the aggressive phenotype of this subtype. Under normal conditions, ZBTB16 acts as a transcriptional repressor that inhibits breast cancer proliferation and metastasis by upregulating ZBTB28 and antagonizing oncogenic factors such as BCL6/ZBTB27 [57]. Elevated ZBTB16 expression has been linked to more favorable prognoses in luminal A breast cancers, where it correlates with higher estrogen and progesterone receptor expression [72]. However, dysregulated m^6^A methylation of ZBTB16 circRNA in TNBC may disrupt this regulatory axis, potentially stabilizing or enhancing translation of oncogenic transcripts rather than mediating tumor suppression. Moreover, recent evidence implicates ZBTB16 in cell cycle control via the ZBTB16/ANXA7/Cyclin B1 pathway, highlighting its broader role in maintaining genomic stability [73]. Thus, aberrant m^6^A modification of ZBTB16 circRNA in TNBC may reprogram its regulatory output, promoting tumor progression and resistance to therapy.

Our finding that METTL8-derived m^6^A circRNA is upregulated in TNBC suggests a potential link between METTL8-driven methylation and the aggressive phenotype of this subtype. METTL8 is a methyltransferase that catalyzes RNA base modifications, including m^3^C and m^6^A, and has been shown to promote cancer cell migration through direct interaction with ARID1A, a key chromatin remodeler [43]. Beyond its role in RNA methylation, SUMOylated METTL8 can induce R-loop formation and genomic instability, driving tumorigenesis [74]. Its methyltransferase activity also extends to mitochondrial tRNAs, linking METTL8 to metabolic regulation and stress adaptation [75]. The observed increase in METTL8 circRNA methylation in TNBC may therefore enhance post-transcriptional regulation that supports cell motility, DNA damage tolerance, and metabolic reprogramming, processes that are integral to the invasive and treatment-resistant nature of TNBC [76].

### 3.3. Genomic Context

Genomic analysis revealed prominent connections centered around regions on chromosomes 1, 3, 11, 12, 15, and 17. The clustering of interactions in these areas suggests the presence of genomic hotspots that may serve as key regulatory hubs. These findings provide insights into the genomic architecture underpinning the observation, although further validation is required to elucidate the functional relevance of these interactions. The clustering of interactions around regions associated with the genes in these areas suggests a potential regulatory network influencing disease progression. Overall, the genomic landscape of m^6^A circRNAs in breast cancer suggests an extensive reprogramming of m^6^A methylation patterns across breast cancer subtypes, with specific genomic loci showing significant hyper- or hypomethylation. These modifications may influence circRNA function and gene regulation, contributing to tumor heterogeneity. Further integration of these methylation profiles with gene expression and clinical data could elucidate the role of m^6^A modifications in breast cancer pathogenesis.

The majority of our newly identified differentially m^6^A-methylated circRNAs in TNBC patients were found within exonic regions, consistent with evidence that m^6^A modifications are enriched in larger exons and often concentrated near upstream and central portions of these regions [32]. Exonic circRNAs typically arise from back-splicing events of mature mRNA exons and are more stable and abundant than other circRNA types due to their covalently closed loop structure [44,77]. Their localization in the cytoplasm allows them to regulate gene expression post-transcriptionally, commonly by acting as microRNA sponges, modulating translation, or interacting with RNA-binding proteins.

Exonic sequences are often involved in key regulatory roles. Therefore, cells may produce more of them to modulate gene expression and maintain homeostasis. The higher expression of exonic circRNAs highlights their importance in gene regulation and their potential as key regulatory molecules. The predominance of exonic circRNAs suggests that m^6^A-mediated regulation of these molecules may play a key role in controlling TNBC-specific gene expression networks through post-transcriptional mechanisms, contributing to tumor aggressiveness and phenotypic diversity. Although it is important to recognize that sequencing technologies or bioinformatics pipelines can be more sensitive to exonic circRNAs, leading to higher apparent expression. While the observed predominance of exonic circRNAs and their m^6^A modification patterns in TNBC suggest a potential m^6^A-mediated regulatory role in subtype-specific post-transcriptional processes, further functional validation, including the evaluation of mRNA targets, is required to determine whether these modifications contribute to TNBC biology.

In contrast, intronic circRNAs (derived from intronic sequences excised during splicing) are often localized in the nucleus, where they influence transcriptional or splicing regulation of their parental genes [77]. Their lower representation in TNBC samples may indicate that exonic circRNAs, particularly those modified by m^6^A, dominate the regulatory landscape in this breast cancer subtype. m^6^A methylation can enhance circRNA stability and translation efficiency or alter their interactions within ceRNA networks [32,46]. Thus, the enrichment of m^6^A marks in exonic circRNAs highlights their potential as critical modulators of TNBC pathophysiology and as promising candidates for biomarker and therapeutic target development, supported by existing evidence and warranting further investigation. Alternatively, the presence of intronic circRNAs might reflect additional layers of post-transcriptional or transcriptional regulation since intronic circRNAs are not simply byproducts but can serve as important regulators within gene expression processes [46].

### 3.4. Clinical Implications of Identified Dysregulated m^6^A circRNAs in TNBC

Building on discoveries of TNBC-specific m^6^A circRNA methylation patterns, recent studies emphasize the potential of targeting ceRNA networks, composed of circRNAs, microRNAs, and mRNAs, as a novel therapeutic strategy. These networks regulate post-transcriptional gene expression and are influenced by epigenetic modifications such as m^6^A, which affect circRNA stability and function. Understanding these interactions may reveal key regulatory nodes that might serve as circulating biomarkers or therapeutic targets. Such strategies could help inhibit metastasis, overcome treatment resistance, and improve survival in TNBC. Integrating m^6^A and circRNA profiling with ceRNA network analysis therefore offers a promising avenue for advancing precision medicine and developing targeted therapies for this aggressive breast cancer subtype.

The identified host genes and associated circRNAs with correlations to survival in TNBC highlight the dualistic roles of m^6^A-modified circRNAs in TNBC prognosis. Oncogenic drivers such as ZBTB16, DOT1L, GSK3B, and VPS18 appear to be supported by hypermethylated circRNAs that may stabilize transcripts, act as miRNA sponges, and sustain pro-tumorigenic pathways like Wnt/β-catenin, VEGF, GTPase signaling, and vesicular trafficking. These interactions have the potential to amplify downstream processes, including epithelial–mesenchymal transition (EMT), invasion, and immune evasion as discussed herein. Indeed, clinical trials have found mutations in DOT1L to be predictive of response to immune checkpoint inhibitors [78]. Likewise, inhibitors of GSK3Β have shown promise in trials as cancer treatments and modulators of anticancer immunity [79]. Conversely, alterations in protective factors, such as ATM, bring to light m^6^A-driven circRNA function that could reinforce DNA repair mechanisms, counteracting genomic instability and improving survival [80].

VPS18 plays a dual role in regulating tumor progression and immune evasion through its functions in vesicular trafficking and receptor regulation. As a component of the homotypic fusion and vacuole protein sorting (HOPS) complex, VPS18 controls lysosomal degradation pathways that influence surface receptor turnover. Recent evidence shows that targeting VPS18 disrupts retromer-mediated trafficking of PD-L1, leading to its lysosomal degradation and enhanced antitumor immune activity, thereby potentiating the effects of immunotherapy [81]. Conversely, VPS18 has also been shown to suppress EGFR expression and lung tumorigenesis, suggesting a context-dependent tumor-suppressive function through modulation of growth factor receptor pathways [82]. Together, these findings position VPS18 as a critical regulator of both tumor cell signaling and immune checkpoint control, implicating its dysregulation in the balance between tumor progression and immune evasion.

Collectively, these findings suggest a complex epitranscriptomic regulatory axis in which m^6^A methylation fine-tunes circRNA expression and function, thereby influencing patient prognosis. The stability and cytoplasmic enrichment conferred by m^6^A marks on oncogenic circRNAs such as hsa_circRNA_103444 (GSK3B) or hsa_circRNA_404935 (ZBTB16) suggest that these molecules could be candidates as circulating prognostic biomarkers or therapeutic targets. Targeting m^6^A writers (e.g., METTL3, METTL14), erasers (e.g., FTO, ALKBH5), or circRNA–miRNA interactions could restore regulatory balance and mitigate tumor aggressiveness. Conversely, preserving beneficial methylation patterns, as observed with ATM and hsa_circRNA_003641, may enhance DNA repair capacity and treatment response. These survival-linked signatures provide a molecular framework for subtype-specific prognostication and precision-targeted therapies in TNBC.

### 3.5. The Significance of Enriched Molecular Pathway Among Host Genes of TNBC-Specific Altered m^6^A circRNAs

Pathway enrichment analysis may inform the identification of novel therapeutic targets. Our enrichment analysis revealed subtype-specific alterations in circRNAs involved in several pathways with importance to breast cancer pathogenesis.

In the comparison between TNBC and luminal tumors, enriched pathways revealed extensive reprogramming of cytoskeletal organization and signal transduction. Activation of Rho GTPase signaling components (RHOA, RAC1, CDC42, RHOG, RAC2) pointed to dynamic regulation of cell motility and intracellular trafficking, while ubiquitin-mediated proteolysis suggested elevated protein turnover and signaling control. Concurrent enrichment in nuclear receptor and androgen receptor pathways, together with altered chromatin remodeling and methyltransferase activity, indicated profound epigenetic shifts influencing gene expression. Furthermore, activation of Wnt/β-catenin, VEGFA, and EPH signaling highlighted proliferative and angiogenic potential, while changes in lipid metabolism and Golgi-to-ER transport underscored metabolic adaptation that supports TNBC’s growth and interaction with its microenvironment.

When comparing TNBC to Luminal B tumors, enrichment in Wnt/β-catenin and signal transduction pathways further emphasized hyperactivation of proliferative and survival mechanisms characteristic of TNBC’s aggressiveness. The presence of mesodermal commitment and focal adhesion signatures reflected enhanced cellular plasticity and metastatic potential, while alterations in Class I MHC-mediated antigen presentation implied immune evasion. Dysregulated calcium signaling and platelet calcium homeostasis suggested microenvironmental communication and signaling crosstalk that may facilitate invasion. Additionally, elevated histone modification and methyltransferase activity confirmed TNBC’s epigenetic reprogramming compared with Luminal B tumors, reinforcing its transcriptional adaptability.

Distinct enrichment patterns between TNBC and Luminal A subtypes highlighted pathways underpinning TNBC’s invasiveness and resilience. Enhanced Rho GTPase signaling, KEAP1–NFE2L2 (NRF2) activation, and SUMOylation supported oxidative stress tolerance and chemotherapy resistance. The enrichment of PPARA and androgen receptor signaling suggested metabolic rewiring independent of hormonal regulation, while non-canonical Hedgehog and neurodegenerative-like stress response pathways further indicated reliance on adaptive survival mechanisms. Collectively, these features define TNBC’s hallmark phenotype of metabolic flexibility, stress resistance, and high metastatic potential.

In contrast, Luminal B versus Luminal A tumors showed upregulation of amino acid and sulfur metabolism, consistent with enhanced biosynthetic and proliferative demands. Activation of ROBO receptor and non-integrin ECM interaction pathways pointed to changes in cellular guidance and adhesion, while signaling through growth factor receptors, MAPK, and second messengers indicated intensified oncogenic signaling. Increased serine/threonine kinase activity and cytoskeletal remodeling reflected a shift toward more proliferative and migratory behavior. Overall, TNBC appears to be dominated by aggressive signaling, epigenetic plasticity, and immune modulation, whereas Luminal B tumors rely more on metabolic and kinase-driven growth programs relative to the less proliferative Luminal A subtype.

### 3.6. circRNAs and Wnt/β-Catenin Signaling in TNBC

Pathway enrichment analysis in our dataset reveals that several upregulated circRNAs, such as those derived from JUP (hsa_circRNA_043602), GSK3B (hsa_circRNA_103444), and ZBTB16 (hsa_circRNA_404935), with fold changes of ~2.04, 2.31, and 3.42, respectively, map to Wnt/β-catenin signaling. The observed upregulation of regulators of GSK3B, a kinase that normally phosphorylates β-catenin for degradation, suggests complex regulatory dynamics that may perturb canonical Wnt signaling [83].

Aberrant Wnt/β-catenin signaling is a defining feature of TNBC, driving tumor proliferation, EMT, and metastasis. Dysregulated circRNAs contribute to this pathway both directly and indirectly. For example, circRNA_069718 has been validated in TNBC to promote proliferation and invasion by sponging miR-558, thereby upregulating the Wnt target gene TCF7L2 [35]. Similarly, circ-ITCH, though more extensively studied in other breast cancer subtypes, functions as a tumor suppressor by stabilizing ITCH-mediated ubiquitination of Dvl proteins, thereby inhibiting Wnt/β-catenin activation [41]. In contrast, predicted circRNAs from genes such as GSK3B and JUP may act as oncogenic regulators in TNBC. Hypermethylation of circRNAs from these loci could impair negative regulation of β-catenin, promoting constitutive pathway activation. Thus, both validated and predicted circRNAs converge on the Wnt axis, reinforcing its central role in TNBC progression.

Moreover, the parallels to CARM1 biology strengthen the mechanistic plausibility: in triple-negative breast cancer, CARM1 promotes proliferation, epithelial–mesenchymal transition (EMT), and stemness, in part by interacting with β-catenin and influencing Wnt pathway targets [84]. Thus, the circRNA patterns in our data may reflect epigenetic and post-transcriptional circuits converging on Wnt/β-catenin signaling, contributing to the aggressive behavior of TNBC relative to luminal subtypes.

### 3.7. circRNAs and CDC42-Mediated Cytoskeletal Remodeling

CDC42, a Rho family GTPase, is pivotal for cytoskeletal dynamics, cell polarity, and invasive potential in TNBC [10]. miRNAs such as miR-136-5p and miR-138-5p, which target CDC42 and other GTPase regulators, have been shown to inhibit invasion and metastasis in breast cancer [85,86]. Altered circRNA methylation in TNBC, including that of DOCK1 (hsa_circRNA_100719), could therefore modify miRNA availability, shifting CDC42 pathway activity toward enhanced migration and invasion.

Subtype-specific differences in circRNA m^6^A methylation in our dataset are enriched for host genes that influence regulation of the CDC42 GTPase cycle. These molecular alterations have important implications for TNBC biology and treatment. Increased circRNA hypermethylation may increase accumulation and sponge tumor-suppressive miRNAs, driving CDC42 activation and metastatic progression, whereas hypomethylation-linked downregulation may release miRNAs to suppress alternative oncogenic targets. Because CDC42 signaling promotes aggressive tumor phenotypes [10], disruption of circRNA–miRNA–CDC42 networks may contribute to TNBC’s poor clinical outcomes. Clinically, circRNAs with distinct methylation profiles could emerge as biomarkers to differentiate TNBC from luminal subtypes or predict metastatic risk. Furthermore, therapeutic strategies that modulate circRNA function or restore miRNA activity represent a potential means of targeting CDC42-driven invasion in TNBC, a subtype that currently lacks effective targeted therapies [9]. It is important to note that the functional relationship between circRNAs and their host genes remains complex and not fully understood. Because circRNA biogenesis does not always correlate with host gene transcription, and m^6^A modification may affect circRNA stability, translation potential, or protein interactions independently of host gene regulation, our observations should not be interpreted as mechanistic. The functional enrichment and prognostic inferences inferred from host gene annotations provide a biological context for the identified circRNAs, but they need further integration with host gene expression analysis and functional assays. Further, integrated analysis of host mRNA expression and methylation could provide further insights into subtype-specific regulatory dynamics.

### 3.8. circRNAs and VEGFA-Mediated Angiogenesis

The VEGFA–VEGFR2 signaling axis is a central driver of tumor angiogenesis and a major clinical target of anti-angiogenic therapies [87]. CircRNAs can act as competing endogenous RNAs (ceRNAs) that sequester anti-angiogenic miRNAs and thereby relieve repression of VEGFA/VEGFR2; this mechanism has been experimentally demonstrated in cancer models. For example, circRNA-MYLK sponges miR-29a to upregulate VEGFA, activating VEGFA/VEGFR2 signaling and promoting angiogenesis in xenograft models [88,89].

In our dataset (Supplementary Table S2), the following circRNAs were differentially methylated: VAV3 circRNA (hsa_circRNA_404557) is hypomethylated; RASA1 (hsa_circRNA_007507) and JUP (hsa_circRNA_043602) are hypermethylated; and DOCK1 (hsa_circRNA_100719) is hypermethylated. These specific circRNA identifiers do not appear to have been previously reported on, to our knowledge. Thus, they should be considered novel observations requiring future validation.

Mechanistically, two validated principles apply: (a) hypermethylated/upregulated circRNAs can increase miRNA sequestration and derepress pro-angiogenic transcripts such as VEGFA, and (b) loss or downregulation of a circRNA that normally sponges anti-angiogenic miRNAs can enhance repression of VEGFA. The circRNA–miRNA–VEGFA/VEGFR2 axis has been repeatedly demonstrated in cancer models [88,89].

Several miRNAs with direct angiogenesis roles strengthen this interpretation. miR-132 functions as an “angiogenic switch” by repressing p120RasGAP, thereby promoting endothelial activation and neovascularization [90]. miR-138-5p inhibits HIF-1α/VEGFA signaling and vascular mimicry in hepatocellular carcinoma and suppresses EMT/migration in breast cancer, indicating its anti-angiogenic role in multiple contexts [85].

Together, the observed hypomethylation of some circRNAs and hypermethylation of others is consistent with mechanisms by which circRNA methylation could modulate VEGFA-VEGFR2 signaling and angiogenesis in TNBC. The direct causal link between the specific dataset IDs and VEGFA activity remains to be validated experimentally (e.g., luciferase sponging assays, circRNA perturbation with VEGFA readouts), but the literature provides a clear mechanistic framework.

### 3.9. Epitranscriptomic Regulation of circRNAs in TNBC

The m^6^A modification landscape profoundly shapes circRNA biology, influencing their stability, nuclear export, and interaction with RNA-binding proteins. Dysregulation of methylation machinery, including writers (METTL3/14), erasers (ALKBH5, FTO), and readers (YTHDF1/2), has been reported in multiple cancers [31,91]. Moreover, histone methyltransferases such as CARM1, SMYD4, and EHMT1 contribute to epigenetic reprogramming [92,93,94]. These modifications can dictate whether circRNAs act as oncogenes or tumor suppressors in TNBC. Understanding the interplay between circRNAs, m^6^A methylation, and host gene networks therefore offers promising avenues for biomarker discovery and therapeutic targeting in this aggressive breast cancer subtype.

Our analyses comparing TNBC with Luminal subtypes reveal subtype-specific shifts in circRNA m^6^A methylation, implicating differential regulation of methyltransferases. Some circRNAs associated with SMYD4, EHMT1, and DOT1L appear under-methylated in TNBC, while others tied to ZCCHC4, METTL8, CARM1, AS3MT, and METTL5 show elevated methylation. These patterns suggest a functional reprogramming of the m^6^A methylation machinery in TNBC.

Among the implicated methyltransferases, CARM1 (PRMT4) is one of the best characterized in breast cancer. CARM1 methylates the SWI/SNF subunit BAF155 at arginine-1064, directing it to oncogenic c-Myc targets and promoting breast cancer metastasis [95]. In a separate study, the same authors demonstrated that CARM1 methylates MED12 and that methylation of MED12 sensitizes breast cancer lines to chemotherapy [96]. These studies confirm that CARM1 modulates chromatin and cellular response pathways via post-translational methylation in breast cancer.

Given these validated roles of CARM1, the TNBC-associated upregulation of circRNAs tied to CARM1 and other methyltransferases may influence circRNA stability, miRNA binding, and downstream gene regulation in vascular growth or metastasis. If the altered methylation states change how circRNAs act as miRNA sponges, that could contribute to the aggressive phenotype of TNBC and represent a path for biomarker discovery or therapeutic development.

### 3.10. Next Steps for Further Research to Delineate the Roles of Identified circRNAs and Their m^6^A Modification

To further elucidate the biological significance of the identified TNBC-specific m^6^A-modified circRNAs, future research should integrate high-resolution mapping of m^6^A sites with functional assays to dissect causal mechanisms. Experimental validation using methylation-sensitive RNA immunoprecipitation followed by sequencing (MeRIP-seq) or LC-MS/MS could confirm the m^6^A modification status of key candidates such as circZBTB16, circDOCK1, and circMETTL8. Functional studies involving CRISPR-based demethylation or knockdown of m^6^A writers and erasers (e.g., METTL3, METTL14, FTO, ALKBH5) could help determine how m^6^A dynamics influence circRNA stability, localization, and interaction with miRNAs and RNA-binding proteins (RBP). In vitro and in vivo models of TNBC should also be leveraged to evaluate how these epitranscriptomic changes affect tumor progression, metastasis, and therapy resistance.

### 3.11. Clinical and Translational Implications

Translational studies should focus on correlating the expression and methylation levels of these circRNAs with patient survival, treatment response, and immune microenvironment features to determine their prognostic and predictive utility. Liquid biopsy-based detection of m^6^A-modified circRNAs could offer noninvasive biomarkers for TNBC diagnosis and disease monitoring. Moreover, computational modeling of circRNA–miRNA–mRNA ceRNA networks incorporating m^6^A-dependent regulation may uncover key regulatory hubs suitable for targeted intervention. Ultimately, integrating multi-omic approaches combining methylome, transcriptome, and proteome data will be essential to define the broader impact of m^6^A-modified circRNAs in TNBC biology and guide the development of epigenetic therapeutics that modulate these pathways for clinical benefit.

The results herein should be interpreted in light of certain limitations, including the cohort size, which may introduce selection bias. Given the limited availability of fresh tissue required for high-throughput RNA sequencing, this study was primarily powered to identify large, biologically robust subtype effects. Therefore, further clinical studies involving larger cohorts are needed to validate these findings. Nonetheless, these large effects are likely to be biologically meaningful, highlighting strongly divergent molecular patterns associated with m^6^A modification of circRNAs.

## 4. Materials and Methods

The study was conducted following the principles of the Helsinki Declaration and received approval from the Clinical Research Committee. All procedures were performed in accordance with institutional guidelines and ethical standards. Clinical and demographic information for each patient was retrieved from electronic medical records, including the Integrated Clinical Information System (ICIS) and PowerChart, under the oversight of the Research Authorization Committee (IRB approval numbers 2210023 and 2160029). The tissue samples were de-identified to protect patient confidentiality and were documented in a secure, restricted-access database.

Fresh breast cancer tissue specimens (TNBC, Luminal B, and Luminal A subtypes), confirmed by a pathologist, were collected from patients undergoing surgical resection at King Faisal Specialist Hospital & Research Centre (KFSHRC). All samples were collected prior to the administration of any adjuvant therapies, including chemotherapy, and were immediately snap-frozen in liquid nitrogen for storage. Patient characteristics are described in Supplementary Table S1.

### 4.1. Sample Preparation and m^6^A RNA Immunoprecipitation (MeRIP) and circRNA Epitranscriptomics

Total RNA was extracted from each sample and quantified using a NanoDrop ND-1000 spectrophotometer (Thermo Fisher Scientific, Waltham, MA, USA). RNA integrity was assessed via Agilent 2100 Bioanalyzer (Agilent, Santa Clara, CA, USA) or MOPS electrophoresis and by qPCR assays to ensure high-quality RNA suitable for downstream analyses, including m^6^A MeRIP. 

Immunoprecipitation of m^6^A-modified RNAs was performed using an anti-m^6^A rabbit polyclonal antibody (Synaptic Systems, Göttingen, Germany, 202003) coupled to Dynabeads M-280 Sheep Anti-Rabbit IgG (Invitrogen, Waltham, MA, USA, 11203D). Briefly, 3–5 μg of total RNA, along with an m^6^A spike-in control mixture, was incubated in 1× IP buffer (50 mM Tris-HCl, pH 7.4; 150 mM NaCl; 0.1% NP40; RNase inhibitor) containing 2 μg of antibody at 4°C with rotation for 2 h. The antibody was immobilized on blocked Dynabeads pre-washed in a 1× IP buffer, and RNA binding was facilitated by incubation at 4°C for an additional 2 h with rotation. Post-incubation, beads were washed thrice with 1× IP buffer and twice with wash buffer (50 mM Tris-HCl, pH 7.4; 50 mM NaCl; 0.1% NP40; RNase inhibitor). Enriched m^6^A-modified RNAs were eluted with an elution buffer containing proteinase K at 50°C for 1 h, followed by acid phenol–chloroform extraction and ethanol precipitation.

Both immunoprecipitated (IP) RNAs and supernatant (Sup) RNAs were treated with RNase R (Epicenter, San Diego, CA, USA) to enrich for circRNAs by degrading linear RNAs. The resulting RNAs were then labeled using the Arraystar Super RNA Labeling Kit (Arraystar, Rockville, MD, USA), with Cy5 for IP samples and Cy3 for Sup samples. Labeled RNAs were purified with RNeasy Mini Kit (QIAGEN, Germantown, MD, USA), and their concentrations and specific activities were measured via NanoDrop ND-1000. Equal amounts (2.5 μg) of Cy3- and Cy5-labeled cRNAs were pooled, fragmented at 60 °C for 30 min with the addition of fragmentation buffer and blocking agent, and hybridized to the Human circRNA Epitranscriptomic Microarray (8 × 15 K, Arraystar, Rockville, MD, USA). Hybridization was performed at 65 °C for 17 h in an Agilent Hybridization Oven. Post-hybridization, arrays were washed, fixed, and scanned using an Agilent G2505C Scanner.

### 4.2. Microarray Data Acquisition and Analysis

Array images were analyzed with Agilent Feature Extraction software (version 11.0.1.1). Raw intensities were normalized using the average of log_2_-scaled spike-in RNA intensities. Only probe signals with Present (P) or Marginal (M) QC flags in at least 4 of 27 samples were retained. The “m^6^A quantity” was calculated based on the normalized IP (Cy5) intensities (raw signals of Cy5-labeled IP RNA are normalized with the average log_2_-scaled Spike-in RNA intensities), reflecting the extent of m^6^A modification, using the formulas:


$${IP}_{Cy5\;normalized\;intensity}=log2\left({IP}_{Cy5\;raw}\right)-Average\left[log2\left({IP}_{spike-in_{Cy5}raw}\right)\right]\;m6A\;quantity\;=\;{IP}_{Cy5\;normalized\;intensity}$$

The percentage of m^6^A modification (%Modified) for a transcript was calculated based on the IP (Cy5-labeled) and Sup (Cy3-labeled) normalized intensities.


$$\%Modified=\;\frac{modified\;RNA}{Total\;RNA}\;=\;\frac{IP}{IP+Sup}\;=\;\frac{{IP}_{Cy5\;normalized\;intensity}}{{IP}_{Cy5\;normalized\;intensity}+{Sup}_{Cy3\;normalized\;intensity}}$$

$${IP}_{Cy5\;normalized\;intensity}=log2\left({IP}_{Cy5\;raw}\right)-Average[log2\left({IP}_{spike-in\_Cy5\;raw}\right)]$$

$${Sup}_{Cy3\;normalized\;intensity}=log2\left({Sup}_{Cy3\;raw}\right)-Average[log2\left({Sup}_{spike-in\_Cy3\;raw}\right)]$$

Normalized intensities were obtained similarly by subtracting the average log_2_ Spike-in intensities from raw signals. Differential m^6^A modification between two groups was evaluated by calculating fold change (FC) and *p*-values via *t*-tests. Thresholds for significance were set at |FC| ≥ 2 and *p* < 0.05. Transcripts meeting these criteria were considered differentially m^6^A-modified. Data were further analyzed to identify candidate circRNAs. All raw array images generated from the Agilent Scanner are available upon request. Both raw *p*-values and Benjamini–Hochberg-adjusted (FDR) q-values were computed. Given the small sample size and resulting limited statistical power, no circRNAs met the significance threshold of FDR < 0.05. Accordingly, differential expression was determined using a combined criterion of |FC| > 2 and raw *p* < 0.05, consistent with typical analytical approaches in circRNA profiling studies.

The standardized effect size (Cohen’s d ≈ 1.45) was translated into a minimum detectable log_2_ fold change (~1.5) using the pooled standard deviation, following established microarray sample-size estimation methods, where effect sizes on the log scale are used to compute detectable fold changes. Post hoc power calculations indicated that our study could detect a minimum log_2_ fold change of ~1.5 (Cohen’s d ≈ 1.45) at 80% power. To retain biologically meaningful candidates and reduce false positives, we applied a more stringent threshold of |FC| > 2 in combination with raw *p* < 0.05.

The distribution of raw *p*-values across all features was examined. The *p*-value histogram demonstrated clear left-tail enrichment, indicating deviation from a uniform null distribution (KS statistic = 0.116, *p* < 2.2 × 10^−16^), and supporting the presence of true biological signal. Permutation-based testing was performed (1000 iterations) to evaluate whether the number of detected differentially expressed circRNAs exceeded random expectation. The empirical *p*-value was calculated as the proportion of permutations yielding an equal or greater number of significant features compared to the observed dataset. The resulting permutation *p*-value (*p* ≈ 0.051) indicates that the detected signal marginally exceeds chance expectations, consistent with true differential regulation but limited by sample size and statistical power.

### 4.3. circRNA Ranking and Volcano Plots

Selection of “top” circRNAs for each comparison was based on the combination of statistical significance and magnitude of change, specifically integrating adjusted *p*-values and fold-change values derived from differential methylation analysis. For each comparison (e.g., TNBC vs. Luminal), differential m^6^A modification levels on circRNAs were assessed using an appropriate statistical test (unpaired *t*-test), yielding *p*-values and fold-change estimates. The *p*-values were adjusted for multiple testing using the false discovery rate (FDR) correction, resulting in adjusted *p*-values (q-values). Only circRNAs with an absolute fold change |FC| > 2 were selected to ensure biological relevance and substantial methylation differences. CircRNAs showing statistical significance (*p* < 0.05) were filtered for further analysis. Among these, the top hypermethylated and hypomethylated circRNAs were ranked by their fold change (FC). The circRNAs with the highest and lowest FC values were selected for downstream analyses. Thus, prioritizing those with the most significant and substantial methylation differences. The discriminatory potential of circRNAs between TNBC and Luminal subtypes was further assessed through Receiver Operating Characteristic (ROC) analysis and the calculation of area under the curve (AUC) values. The ROC curves were generated using the pROC (version 1.18.5) and ROCR (version 1.0-11) packages in R.

### 4.4. Annotation of circRNA/miRNA Interactions

Predicted circRNA/miRNA interactions were identified using TargetScan (version 8.0) and miRanda (version 3.3a) software. Differentially m^6^A-modified circRNAs were annotated with their potential miRNA binding partners, emphasizing those with known roles in cancer. The predicted circRNA/miRNA interaction predictions were obtained from Arraystar’s miRNA target prediction software based on TargetScan and miRanda. Biological, functional and pathway-level annotations of the circRNA host gene were obtained from the DAVID tool.

### 4.5. Functional Enrichment Analysis

Functional enrichment analysis was performed using the Database for Annotation, Visualization and Integrated Discovery (DAVID) to identify significantly enriched biological pathways associated with the differentially modified host genes of circRNAs. Enrichment results were filtered to retain only terms with an adjusted *p*-value < 0.05. To visualize the functional enrichment patterns, the GOplot (version 1.0.2) package [97] in R was used. The log_2_ fold change (log_2_ FC) values of circRNAs derived from the subtype-specific comparisons (e.g., TNBC vs. Luminal tumors) were mapped to enriched functional terms of their host genes, and the associations were visualized as heatmap.

### 4.6. Survival Analysis

Survival analysis was performed using the R packages survival (version 3.7-0) and survminer (version 0.4.9) [98]. A survival object was constructed from patient data, where overall survival time (in days) was used as the time variable and survival status (alive = 0, dead = 1) as the event indicator. Kaplan–Meier (KM) curves were generated to estimate overall survival as well as survival stratified by clinical subtypes (Classifications) and tumor grade. The log-rank test was applied to assess statistical differences between groups. In addition, cumulative hazard plots were generated to visualize hazard accumulation over time, both for the entire cohort and across subtypes. Risk tables were incorporated alongside selected KM curves to display the number of patients at risk at different time points.

**Figure 10:**
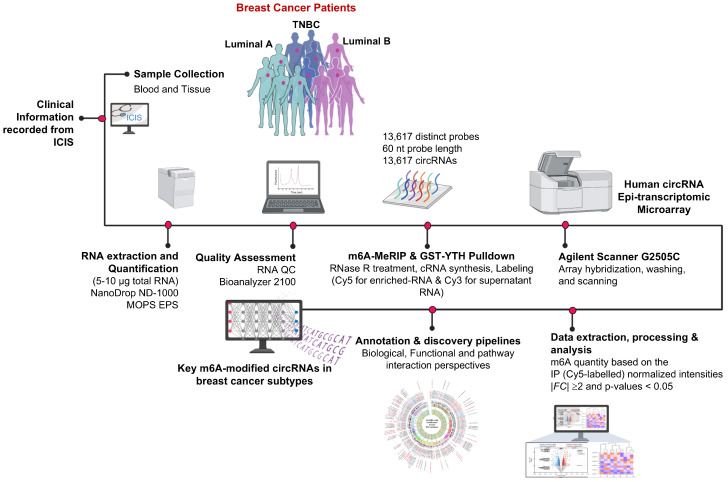
A schematic illustrating the sequence of work to identify key m^6^A-modified circRNAs in breast cancer subtypes, integrating epitranscriptomics and functional pathway analysis.

The prognostic significance of the host genes of m^6^A-modified circRNAs was assessed using the current breast cancer analysis platform of Kaplan–Meier Plotter (https://kmplot.com/analysis/index.php?p=service&cancer=breast; assessed on 9 September 2025), an online database that has the integrated gene expression and survival analysis options from multiple publicly available transcriptome profiles. The current breast cancer analysis platform of KMPlotter provides information on a total of 4929 breast cancer patients, including various molecular subtypes of breast cancer. Thus, using the platform, the circRNA host gene’s prognostic significance was analyzed at the subtype level, especially for TNBC, by restricting the analysis to ER, PR, and HER2 negative-status samples. In total, 392 TNBC patients with RFS information and 153 samples with OS information were used for the analysis. For each gene, patients were split into high and low expression groups based on the default median-based approach and JetSet best probeset method. Kaplan–Meier survival curves were generated for OS or RFS, restricting the analysis to only the specific subtypes of breast cancer. Hazard ratios (HRs) with 95% confidence intervals (CIs) were estimated using a univariate Cox proportional hazards regression model. Log-rank test *p*-values were used to evaluate statistical significance between survival distributions of expression groups. All *p*-values < 0.05 were considered statistically significant. The overall workflow is described in Figure 10.

## 5. Conclusions

This study indicates distinct subtype-specific m^6^A methylation patterns in circRNAs, revealing subtype-dependent epitranscriptomic regulation with diagnostic and prognostic potential biomarkers. Key circRNAs, including circZBTB16, circDOCK1, and circMETTL8, demonstrated significant differential methylation in TNBC compared to luminal subtypes and were associated with pathways linked to tumor proliferation, invasion, and immune modulation. These findings suggest that m^6^A-modified circRNAs contribute to the aggressive phenotype of TNBC and may serve as novel biomarkers or therapeutic targets, which are potential scenarios that justify further research. Collectively, this work establishes a foundation for further mechanistic and translational studies aimed at exploiting circRNA methylation dynamics to advance precision oncology in triple-negative breast cancer (TNBC and mTNBC).

## Data Availability

The datasets generated and/or analyzed during this study are not publicly available due to institutional and patient privacy restrictions. However, the data can be made available from the corresponding author upon reasonable request and with appropriate institutional approvals.

## Supplementary Materials

The following supporting information can be downloaded at https://www.mdpi.com/article/10.3390/ijms27010529/s1.
