# Supplementary material for: Subtype-Specific m6A circRNA Methylation Patterns Identify Epigenetic Biomarker Candidates of Potential Diagnostic and Prognostic Significance in Breast Cancer
Source: Int J Mol Sci. 2026 Jan 4;27(1):529. doi: 10.3390/ijms27010529 (PMC12786885; doi:10.3390/ijms27010529)
Supplement: Supplementary file 1 [file ijms-27-00529-s001.zip › 2026 Dr. Amal Qattan. All rights reserved. Supplementary Figures m6A Methylation Epigenetic Biomarkers in Breast Cancer..pdf]

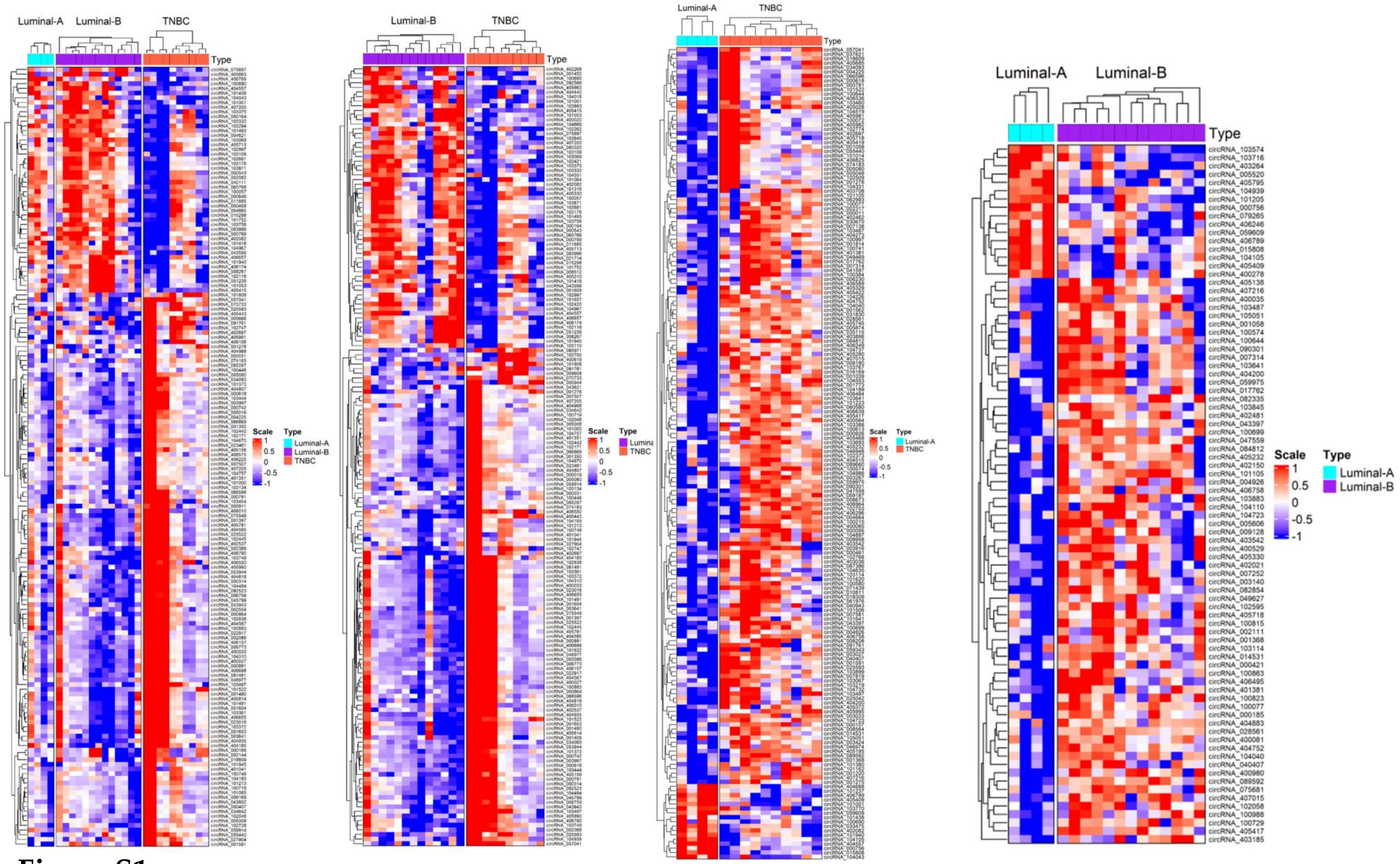

Supplementary Figure S1:

Heatmap showing the methylation patterns of differentially m⁶A-modified circRNAs across breast cancer subtypes.

The heatmaps depict the methylation pattern of circRNAs that exhibited significant differential m6A modifications among the comparisons **A)** TNBC Vs Luminal Tumors, **B)** TNBC Vs Luminal B tumors, **C)** TNBC Vs. Luminal A and **D)** Luminal B Vs Luminal A tumors respectively. Each row represents individual circRNA, and columns correspond to tumor samples grouped by breast cancer subtype. Color intensity reflects the relative m⁶A methylation level, with red indicating higher methylation and blue indicating lower methylation. Hierarchical clustering of both circRNAs and samples was performed to highlight subtype-specific methylation patterns. Significantly hyper or hypo methylated circRNAs with the significant p-value range < 0.05 and $|FC| > 2$ were considered differentially expressed. © 2026 Dr.Amal Qattan. All rights reserved.

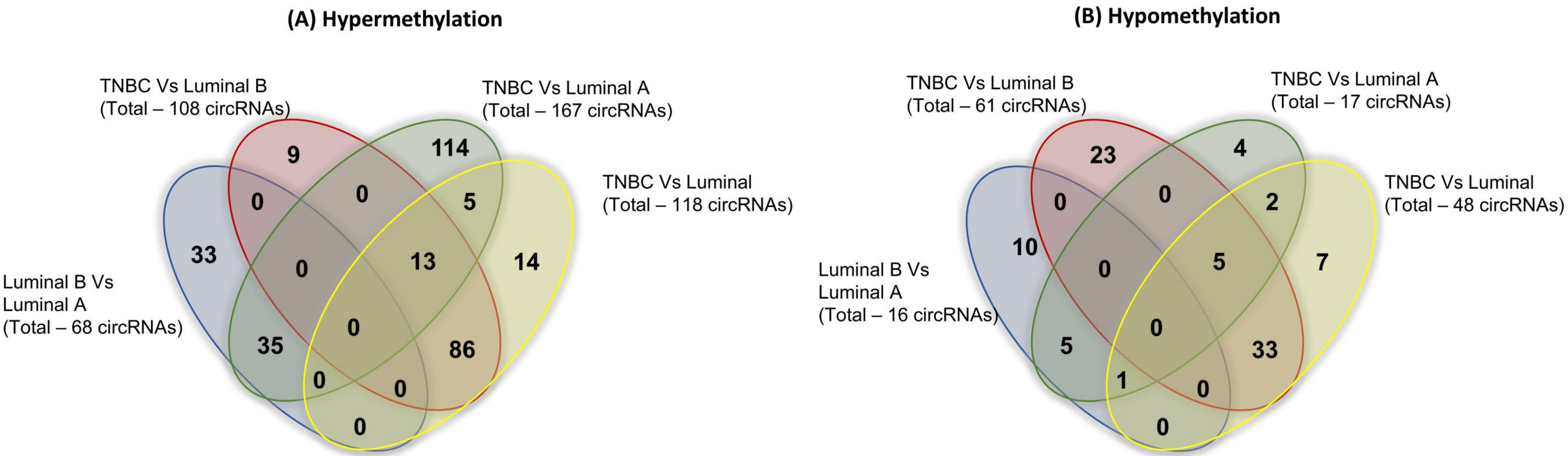

Supplementary Figure S2:
Venn diagram illustrating the overlap of differentially m6A-modified circRNAs across breast cancer subtype datasets: (A) Hypermethylation and (B) Hypomethylation between TNBC vs. all luminal types, TNBC vs. Luminal A, TNBC vs. Luminal B, and Luminal A vs. Luminal B comparisons. The diagram shows the number of unique and commonly identified circRNAs in each comparison group.
© 2026 Dr.Amal Qattan. All rights reserved.

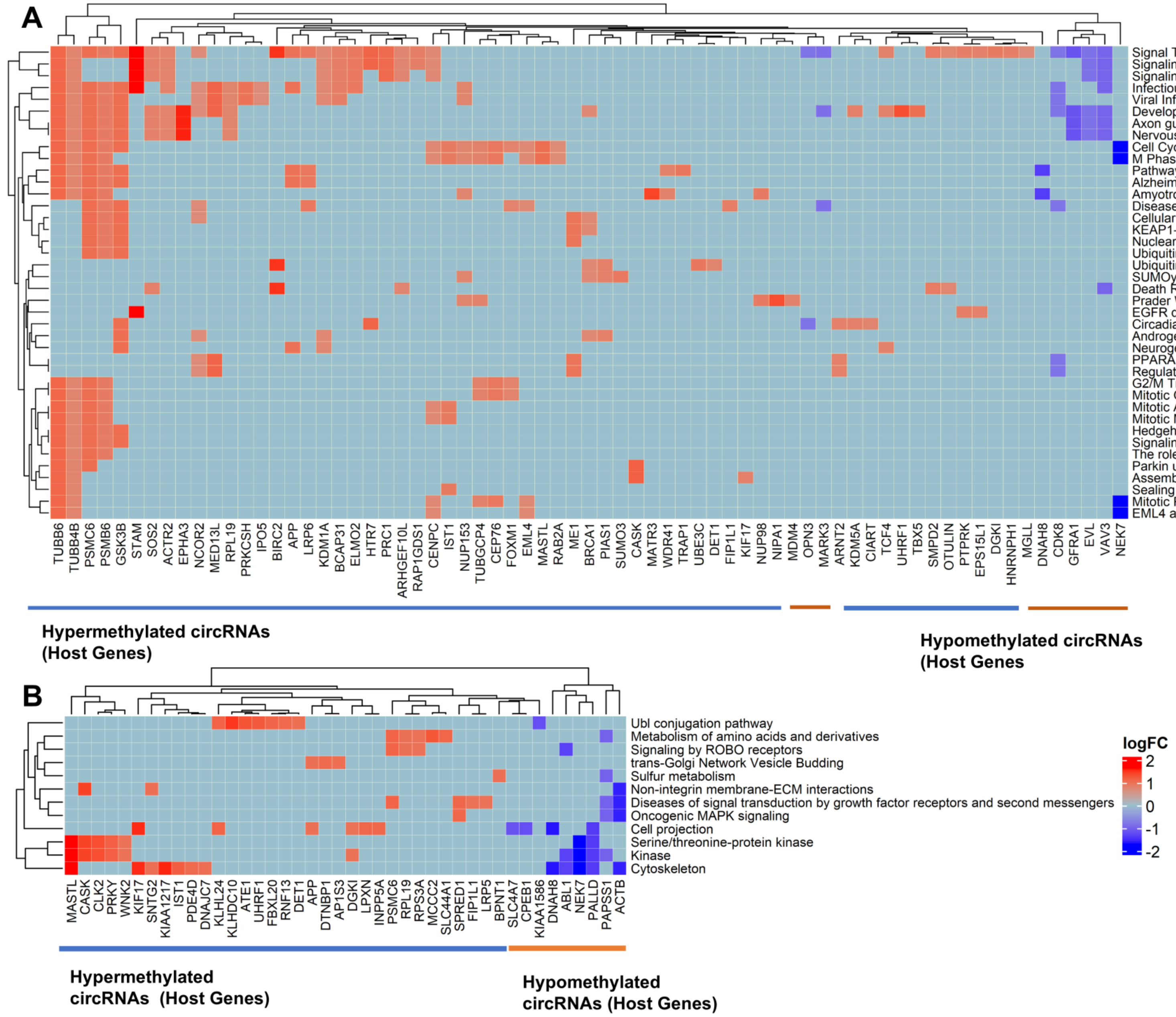

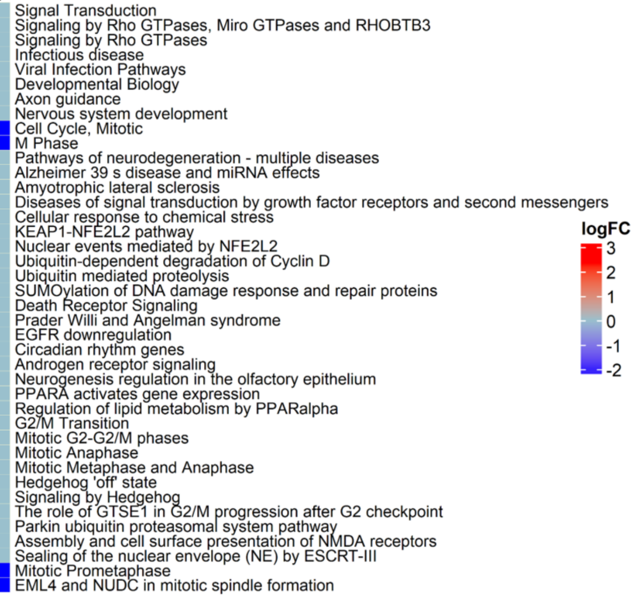

Supplementary Figure S3:
Enrichment analysis of host genes for differentially m^6A-modified circRNAs. Host genes corresponding to differentially m^6A-modified circRNAs were identified and DAVID functional annotation analysis was performed to determine significantly enriched (p-value < 0.05) biological and functional terms. Using the R package GOplot, the relationships between enriched terms and associated host genes of circRNAs are visualized as a heatmap for all three comparison (**A)** TNBC vs. Luminal A tumors, (**B)** Luminal B vs. Luminal A tumors. Each column in the plot represents the log_2 fold change (logFC) of a circRNA with their corresponding host gene name in x-axis. The red color in the heatmap, indicates higher logFC (hypermethylated) and blue indicates lower logFC (hypomethylated). Light blue indicates the absence of a gene for a given term (no logFC). Hierarchical clustering, groups genes with similar annotations and methylation patterns, highlighting functionally related sets of hyper- and hypomethylated circRNAs.

© 2026 Dr.Amal Qattan. All rights reserved.

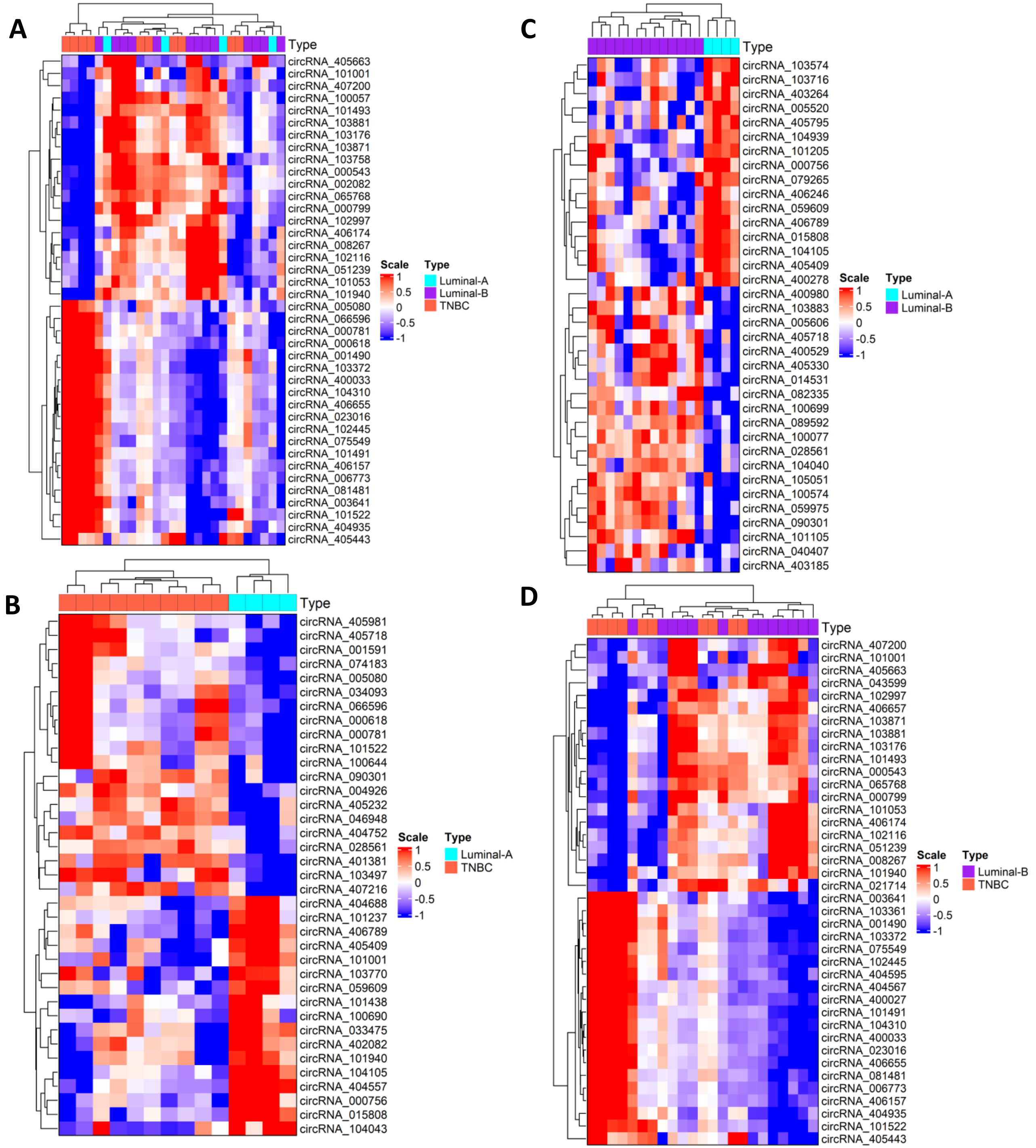

Supplementary Figure S4:
Unsupervised hierarchical clustering of differentially methylated circRNAs across breast cancer subtypes.
(A–D) Unsupervised hierarchical clustering heatmaps showing the top 20 differentially methylated circRNAs identified in each pairwise comparison **(A)** TNBC vs. Luminal tumors, **(B)** TNBC vs. Luminal A tumors, **(C)** Luminal A vs. Luminal B tumors, and **(D)** TNBC vs. Luminal B tumors. For each comparison, unsupervised hierarchical clustering was performed at the sample level based on the circRNA m^{6}A methylation patterns. As expected, due to the intrinsic heterogeneity of TNBC, clusters in panel **A & D** show partial intermixing of TNBC with Luminal subtypes. In contrast, panels **B and C** exhibit clearer subtype separation under fully unsupervised conditions.

© 2026 Dr.Amal Qattan. All rights reserved.
